# Functional analysis of a novel endo-β-1,6-glucanase *Mo*Glu16 and its application in detecting cell wall β-1,6-glucan of *Magnaporthe oryzae*

**DOI:** 10.3389/fmicb.2024.1429065

**Published:** 2024-07-04

**Authors:** Yanxin Wang, Ding Li, Zhoukun Li, Zhongli Cui, Xianfeng Ye

**Affiliations:** ^1^College of Life Sciences of Liaocheng University, Liaocheng, China; ^2^Key Laboratory of Agricultural Environmental Microbiology, Ministry of Agriculture and Rural Affairs, College of Life Sciences of Nanjing Agricultural University, Nanjing, China; ^3^Jiangsu Academy of Agricultural Sciences, Institute of Veterinary Immunology & Engineering, Nanjing, China

**Keywords:** β-1,6-glucanase, *Magnaporthe oryzae*, specificity, β-1,6-glucan, cell wall

## Abstract

As an essential component of the fungal cell wall, β-1,6-glucan has an important role in the growth and development of fungi, but its distribution has not been investigated in *Magnaporthe oryzae*. Here, a novel β-1,6-glucanase from *M. oryzae*, *Mo*Glu16, was cloned and expressed in *Pichia pastoris*. The enzyme was highly active on pustulan, with a specific activity of 219.0 U/mg at pH 5.0 and 50°C, and showed great selectivity for continuous β-1,6-glycosidic bonding polysaccharides. Based on this, β-1,6-glucan was selectively visualized in the vegetative hyphae, conidia and bud tubes of *M. oryzae* using a hydrolytically inactive GFP-tagged *Mo*Glu16 with point mutations at the catalytic position (His-*Mo*Glu16^E236A^-Gfp). The spore germination and appressorium formation were significantly inhibited after incubation of 10^5^/ml conidia with 0.03 μg/μl *Mo*Glu16. Mycelia treated with *Mo*Glu16 produced reactive oxygen species and triggered the cell wall integrity pathway, increasing the expression levels of genes involved in cell wall polysaccharide synthesis. These results revealed that *Mo*Glu16 participated in the remodeling of cell wall in *M. oryzae*, laying a foundation for the analysis of cell wall structure.

## Introduction

*Magnaporthe oryzae* is a devastating hemibiotrophic fungus (Dean et al., 2012) that attacks more than 50 different plant species (Langner et al., 2018), resulting in a losses of up to 30% of grain production (Wilson and Talbot, 2009) and posing a serious threat to global food security. So far, the chemical fungicides are commonly used to the control of rice blasts, such as tricyclazole inhibiting the melanization of appressoria (Zhou et al., 2022); however, the long-term use of chemical pesticides leads to a series of serious problems, such as environmental pollution, the destruction of farmland ecology and natural ecosystems, the increase of pathogen drug resistance and the fungicide residue in food. Thus, it is urgently required to develop a safer, more efficient and more reliable antifungal strategies to control rice blast fungus. The cell wall is the outer barrier of *M. oryzae* to resist external stress, including inappropriate pH, osmotic stress, temperature changes, oxidative stress and antifungal drugs, moreover, it enables to maintain cellular integrity and viability (Lu K. et al., 2023). Thus, overcoming the fungal outer barrier is crucial in the development of biocontrol strategies to prevent pathogenic infection. Analyzing the cell wall components is conducive to understanding the structure of the cell wall and the mechanism of its biogenesis in *M. oryzae*, providing a crucial foundation for the development of novel antifungal interventions.

The fungal cell wall is comprised mainly of α- and β-glucans, along with chitosan, chitin and glycosylated proteins (Fontaine et al., 2000; Aimanianda et al., 2009). At present, the distributions of major polysaccharides including α-1,3-glucan, β-1,3-glucan, chitin, chitosan in the conidia, germ tubes, appressoria, and infection hyphae of *M. oryzae* have been revealed by fluorescent labeling and imaging techniques (Fujikawa et al., 2009). Recently, Li et al. (2019) reported β-1,6-glucan was also the essential component in cell wall of *M. oryzae*, however, its distribution in blast rice was not studied. β-1,6-glucan widely presents in various fungi (Li et al., 2023), especially in *Malassezia restricta*, it comprises approximately 93% of the total glucan in the cell wall and acts as the dominating structural element (Stalhberger et al., 2014). As a fundamental tie between cell wall proteins and the carbohydrates (Pan et al., 2017), β-1,6-glucan enables mannoproteins to join the β-1,3-glucan and chitin-based structural network, as well as covalently links with the C-terminal of glycosyl phosphatidylinositol (GPI)-anchored proteins (Ye et al., 2023), which maintains the cell wall integrity and is crucial in the fungal growth and morphogenesis. In *Colletotrichum graminicola*, knockdown of genes involved in synthesis of β-1,6-glucan resulted in swelling of necrotrophic hyphae, rupture of appressoria, and decline of appressorial adhesion (Oliveira-Garcia and Deising, 2016). Moreover, decreased levels of β-1,6-glucan in the cell wall also inhibited hyphal formation in *Candida albicans* (Umeyama et al., 2006). In addition, β-1,6-glucan has been found only in the cell wall of fungi and oomycetes (Fesel and Zuccaro, 2016), indicating that it may be the potential target in the biological control to plant fungal pathogens.

Beta-1,6-glucanases, also named as pustulanases, cleave the β-1,6-glycosidic bond of β-1,6-glucan via exo- or endo-type mechanisms, generating oligosaccharides and glucose (Temple et al., 2017). Based on the sequence conservation of the amino acid residues located in the catalytic domain, all currently reported β-1,6-glucanases were categorized into glycoside hydrolase families 5 and 30 (Volkov et al., 2021). The majority were found in fungi, such as GH30A from *Coprinopsis cinerea* (Liu et al., 2020), LePus30A from *Lentinula edodes* (Konno and Sakamoto, 2011), β-1,6-glucanase from *Aspergillus fumigatus* (Boisramé and Gaillardin, 2009) and GcnA from *Epichloë festucae* (Bryant et al., 2007). By contrast, only a few β-1,6-glucanase were found in bacteria, such as PsGly30A from *Paenibacillus* sp. GKG (Plakys et al., 2024), CpGlu30A from *Chitinophaga pinensis* (Lu Z. et al., 2023) and Gly30B from *Saccharophagus degradans* 2–40^T^ (Wang et al., 2017). In addition, β-1,6-glucanases were reported to be involved in mycoparasitism, autolysis and extensibility of fungal cell wall. de la Cruz et al. (1995)reported that *Trichoderma* secreted the extracellular β-1,6-glucanases digesting host cell walls to facilitate their mycoparasitism. Similarly, Li et al. (2019) found that *Corallococcus* sp. produced a β-1,6-glucanases hydrolyzing fungal cell walls to prey on the rice blast. Moreover, Konno and Sakamoto (2011) detected that, in *Lentinula edodes*, a β-1,6-glucanases LePus30A had higher expression in fruiting bodies preserved for 3 days after harvest than in fresh fruiting bodies, and proposed that LePus30A was involved in cell wall degradation during autolysis of the fruiting body. Furthermore, Liu et al. (2020) discovered that, in *Coprinopsis cinerea,* a endo-β-1,6-glucanases GH30A showed higher expression in areas of the basal stipe that are not growing or that are swollen at the base, revealing the crucial role of GH30A in inhibiting basal stipe elongation and growth. However, little information is known regarding the biological function of the β-1,6-glucanase from *M. oryzae*.

In this study, a novel β-1,6-glucanase *Mo*Glu16 from *M. oryzae* was successfully expressed in *Pichia pastoris*. Conidial germination and appressorium formation are two key steps in the disease cycle of *M. oryzae*, in which the cell wall was reconstructed in order to successfully infect the host cell. Here, to determine the biological function of *Mo*Glu16 in the growth of *M. oryzae*, we also detected its effect on mycelium and conidia development of rice blast.

## Material and method

### Strains and plasmids

*Magnaporthe oryzae* 70–15 (taxonomy ID: 242507) stored in our laboratory was used to amplify the β-1,6-glucanase gene *MoGLU16* (GenBank accession: MGG_14602). *Escherichia coli* DH5α was used for plasmid storage and amplification. *Pichia pastoris* GS115 purchased from Invitrogen was used to heterologously express the β-1,6-glucanase *Mo*Glu16. The pEFαA vector backbone containing bleomycin (Zeocin) resistance was used to construct the recombinant plasmid. All strains and plasmids described above are preserved in our laboratory.

### Analysis of the sequence and structure of MoGlu16

On the basis of the amino acid sequences of *Mo*Glu16 and other reported β-1,6-glucanases in which contained six representative members from GH30 family and GH5 family, respectively (Plakys et al., 2024), a phylogenetic tree of *Mo*Glu16 was constructed using the neighbor-joining method in MEGA v.6.06 software with 1,000 bootstrap iterations. The amino acid sequences of the above thirteen proteins extracted from GH30 and GH5 family were from NCBI. Sequence conservation of *Mo*Glu16 and other β-1,6-glucanases was determined on the basis of amino acid sequences alignment in ClustalW.

I-TASSER was used to predict the three-dimensional structure of *Mo*Glu16 (Roy et al., 2010). Among five structures generated by I-TASSER, the models with the highest C-score were selected to accurately analyze by TM-align.^1^ Visualization and analysis of the models were conducted using PyMOL software (DeLano Scientific LLC, San Carlos, CA).

### RNA extraction and cDNA synthesis

RNA of the *M. oryzae* was extracted from mycelia cultured by CM liquid cultures, grown at 28°C, 180 rpm for 36 h. Extraction was performed using a Spin Column Fungal Total RNA Purification Kit (Sangon, China) following the manufacturer’s instruction for purification of total RNA. cDNA synthesis was conducted using a HiScript II Q Select RT SuperMix for qPCR (+gDNA wiper) Kit (Vazyme Biotech Co., Ltd., China) according to the manufacturer’s instruction.

### Gene cloning and construction of expression plasmids

The amino acid sequence of *Mo*Glu16 (GenBank accession: XP_ 003708898.1) was derived from the *M. oryzae* 70–15 genome at NCBI. The signal peptide was predicted using the Signal P4.1 server. The cDNA sequence encoding the *Mo*Glu16 protein without the signal peptide was amplified using the primer pair *Mo*Glu16-F/*Mo*Glu16-R (Supplementary Table S1) generated by Primer Premier 5 software. To obtain the recombined plasmid pEFαA-*Mo*glu16-6His, the above PCR amplified product (the cDNA of *Mo*Glu16) was purified and ligated into the plasmid pEFαA digested with the restriction enzymes (*Xho* I and *Xba* I) using the ClonExpress™ II/One Step Cloning Kit (Vazyme Biotech Co., Ltd., China).

### Expression and purification of recombinant proteins

The yeast spheroplasts were prepared according to the manual mentioned in the *Pichia* Expression Kit (Yamunasri et al., 2021). In brief, *P. pastoris* was cultured at 28°C and 180 rpm, the cells were harvested when OD_600_ reached 0.2 to 0.3, the pellets were resuspended in the solution containing 100 mM Tris, 0.6 M sorbitol, 100 mM dithiothreitol (DTT) and 100 mM lithium acetate, left for 30 min at 28°C, then centrifuged at 4500 g, 4°C for 10 min. Finally, the pellets were washed three times with 10 mL of 1 M sorbitol and were resuspended in 500 μL sorbitol. The yeast transformation was conducted according to the method reported by Wu and Letchworth (2004). *P. pastoris* spheroplasts were electroporated with 3 μg of the above recombinant plasmids pEFαA-*Mo*glu16-6His linearized by *Cla* I. Transformants were selected on YPD plates with 100 μg/mL Zeocin, screened by colony PCR with the primer pair (5-α-factor/3-AOX) (Supplementary Table S1) and confirmed by Sanger sequencing. The YPD plates contained 10 g/L yeast extract, 20 g/L peptone, 20 g/L dextrose and 15 g/L agar.

The positive transformants were cultured at 30°C, 220 rpm for 12–16 h in 15 mL BMGY medium containing 10 g/L yeast extract, 20 g/L peptone, 13.4 g/L yeast nitrogen base, 10 g/L glycerol, 0.4 mg/L biotin and 100 mM potassium phosphate (pH 6.0). The yeast cells were obtained by centrifuging at 12000 *g*, 4°C for 10 min, and transferred into 100 mL BMMY medium containing 10 g/L yeast extract, 20 g/L peptone, 13.4 g/L yeast nitrogen base, 0.5% methanol, 0.4 mg/L biotin and 100 mM potassium phosphate (pH 6.0), followed by culture at 30°C and 220 rpm. To induce the recombined protein expression, 0.5% methanol was added to the above medium every 24 h. After 6 days, the yeast fermentation broth was centrifuged at 12000 *g*, 4°C for 30 min to collect the supernatant. The recombined protein in the supernatant was purified using Ni^2+^-nitrilotriacetic acid (NTA) resin (88,221, Thermo Fisher Scientific, USA) following the manufacturer’s instructions. The purity and molecular size of purified *Mo*Glu16 were assessed by sodium dodecyl sulfate-polyacrylamide gel electrophoresis (12% SDS-PAGE) with Coomassie G-250 staining. The concentration of the purified protein was analyzed using the Bradford method (Niu et al., 2016).

### *Mo*Glu16 activity assay

The amount of reducing sugars produced by *Mo*Glu16 from pustulan or similar polysaccharides was estimated by the 3,5-dinitrosalicylic acid (DNS) (Wood et al., 2012). The 200 μL reaction solutions containing 0.2 μg purified *Mo*Glu16 and 0.5% (w/v) polysaccharides (pustulan, yeast glucan, laminarin, *M. oryzae* cell wall, pachymaran, corncob or beech wood xylan) in 50 mM sodium citrate (pH 5.0), were incubated for 30 min at 40°C. Then, 200 μL of DNS reagent was added and boiled for 10 min. To measure the amount of reducing sugar in solution, the above reaction mixtures were centrifuged and the absorbance of the supernatant at 540 nm was recorded. Each treatment was replicated three times in the above experiments. The amount of *Mo*Glu16 required to produce reducing sugars equal to 1 μmol of glucose per minute was defined as one unit of glucanase activity.

To assay the effect of pH on hydrolytic activity, different pH (3.0–11.0) buffers with concentrations of 50 mM, including pH 3.0–6.0 sodium citrate buffer, pH 6.0–7.0 potassium phosphate buffer, pH 7.0–9.0 Tris–HCl buffer and pH 9.0–11.0 glycine-NaOH buffer, were used in the *Mo*Glu16 activity assays. To measure the effect of temperature on the action of *Mo*Glu16 on pustulan, the reaction solutions were placed at 20–80°C for 1 h. In order to test the stability of *Mo*Glu16, the mixtures were incubated at pH 3.0–11.0 for 12 h or at 20–80°C for 1 h without the substrate, then contacted with the substrate pustulan at pH 5.0 and 50°C to detect the remaining activity of *Mo*Glu16 under the reaction conditions described above. To determine activity changes of *Mo*Glu16 in the presence of metal ions or the chelating agent EDTA, *Mo*Glu16 was incubated in 50 mM pH 5.0 sodium citrate buffer containing the indicated metal salts (1 mM) or EDTA (1 mM and 10 mM) at 4°C for 1 h. Then, the substrate pustulan was added to assay the remaining activity at 50°C for 1 h. Three replicates of each treatment was conducted in all of the above experiments.

### Analysis of *Mo*Glu16-generated pustulan hydrolysate

Reaction mixtures comprising 0.2 μg *Mo*Glu16 and 200 μL pustulan (0.5%, w/v) were incubated at 50°C for 5 min, 15 min, 30 min, 1 h, 2 h, 4 h, 8 h and 12 h, respectively. Then, the mixtures were boiled for 10 min and centrifuged for 1 min at 12000 rpm to remove the insoluble substances. The distribution of hydrolysate components in the supernatant was assessed using thin-layer chromatography (TLC), and their molecular mass was evaluated by matrix-assisted laser desorption ionization-time-of-flight mass spectrometry (MALDI-TOF MS). The TLC detection and mass spectrometry identification of hydrolysate components were carried out following the method reported by Wang et al. (2021a). In a nutshell, the TLC plate added the hydrolysate was developed in the solvent including ethyl acetate/acetic acid/water (3,2:1, v/v/v), then sprayed with 0.5% thymol (dissolved in ethyl alcohol containing 5% sulfuric acid) and heated at 95°C to visualize the products. In mass spectrometry detection, 1 μL of the 2,6-dihydroxybenzamide (DHB) droplet (20 mg/mL DHB dissolved in 30% acetonitrile with 0.1% trifluoroacetic acid (TFA)) was applied to a ground-steel plate (MFP 384 ground-steel target plate TF; Bruker Daltonics, Bremen, Germany), and 1 μL of the hydrolysate was mixed into the DHB droplet and dried under a steam of air, the mixture was spontaneously crystallized. A nitrogen laser (337 nm, 3 ns pulse width, 3 Hz) was used for ionization and aimed either at the central area of the sample or at the outmost edge of the crystal rim. Mass spectra (MS) acquired over the scan range m/z 100–2000 was recorded on a Bruker Reflex II (Bruker Daltonics, Bremen, Germany) in the positive ion mode.

### Detection of β-1,6-glucan in the *Magnaporthe oryzae* by the derivant of *Mo*Glu16

The mutant derivatives (designated as *Mo*Glu16^E236A^ and *Mo*Glu16^E332A^) of *Mo*Glu16 were designed based on the analysis of conserved domains, and constructed using the Mut Express II Fast Mutagenesis Kit V2 (Vazyme Biotech Co., Ltd., China), with recombinant plasmid pEFαA-*Mo*glu16-6His as template and corresponding oligonucleotides (E236A-F/E236A-R and E332A-F/E332A-R) (Supplementary Table S1) as primers. The transformed strains of *E. coli* containing the recombinant plasmids (pEFαA-*Mo*glu16 ^E236A^-6His or pEFαA-*Mo*glu16 ^E332A^-6His) were verified by Sanger sequencing. The plasmids (pEFαA-6His-*Mo*glu16^E236A^-GFP and pEFαA-*Mo*glu16^E226A^-GFP-6His) encoding the fluorescent derivatives (His-*Mo*Glu16^E236A^-Gfp and *Mo*Glu16^E236A^-Gfp-His) were constructed using sequences from pEFαA-*Mo*glu16^E236A^-6His and GFP as templates, and corresponding oligonucleotides (N-His-F1/NC-His-R1, NC-His-F2/N-His-R2; C-His-F1/NC-His-R1, NC-His-F2/C-His-R2) (Supplementary Table S1) as primers. The above recombinant plasmids linearized by *Cla* I were transferred to *P. pastoris* spheroplasts by electroporation. The positive transformants of *P. pastoris* selected by colony PCR were cultured on BMMY medium and induced by 0.5% methanol to obtain the corresponding target proteins.

Preparation, collection and development of conidia of *M. oryzae* were carried out as described before Wang et al. (2021b). Vegetative hyphae, conidia, bud tubes and appressoria of *M. oryzae* were incubated with 20 μg His-*Mo*Glu16^E236A^-Gfp at 28°C for 1 h, respectively, then rinsed three times with 20 mM Tris–HCl buffer (pH 7.0), and examined using the fluorescence microscope (CLSM, Leisa, TCSSP2). All of the above experiments were repeated three times.

### Effect of *Mo*Glu16 on mycelia and spore development

To assay the effect of *Mo*Glu16 on conidia of *M. oryzae*, the conidia were treated with *Mo*Glu16 (0, 0.15, 0.20, 0.25, and 0.30 μg/μl) and placed on Gel Bond film (FMC Bioproducts, Rockland, ME) at 28°C. The development of conidia was examined after 4 and 8 h using differential interference contrast (DIC) microscopy. Three replicates of every treatment were performed in all of the above experiments.

The effect of *Mo*Glu16 on the mycelium of *M. oryzae* was assessed by detecting the production of reactive oxygen species (ROS), the integrity of the cell membrane and the distribution of chitin. The ROS were detected as described by Zhou et al. (2019). Mycelium of *M. oryzae* cultured on the liquid media was collected and incubated with 0.30 μg/mL *Mo*Glu16 for 12 h at 28°C, rinsed with 20 mM Tris–HCl (pH 7.0), then treated for 15 min at 25°C. The ROS production was detected by 2,7-dichlorodihydrofluorescein acetoacetic acid (H_2_DCFDA) (50 μM), the cell membrane integrity was monitored by propidium iodide (PI) (5 μg/mL), the chitin distribution in the mycelium was measured by staining with calcofluor white (CFW) (10 mg/mL). All images were observed under confocal laser-scanning microscope (CLSM, Leisa, TCSSP2). In addition, the transcription levels of genes involved in ROS production and cell wall polysaccharide synthesis were detected in mycelia treated with *Mo*Glu16 or inactivated *Mo*Glu16. Genes associated with ROS production contained glyoxalase gene (MGG_02069), peroxidase genes (MGG_04404, MGG_04545 and MGG_13239), the superoxide-generating NADPH-oxidase genes (*NOX1*, MGG_00750; and *NOX2*, MGG_06599) and oxidative stress regulator gene (*YAP1*, MGG_12814); their expression levels were examined by the primers (020-QF/020-QR, 044-QF/044-QR, 045-QF/045-QR, 132-QF/132-QR, NOX1-QF/NOX1-QR, NOX2-QF/NOX2-QR and YAP-QF/YAP-QR) (Supplementary Table S1). Genes involved in cell wall polysaccharide synthesis contained seven chitin synthase genes (*MoCHS1*, MGG_01802; *MoCHS2*, MGG_04245; *MoCHS3*, MGG_09551; *MoCHS4*, MGG_09962; *MoCHS5*, MGG_13014; *MoCHS6*, MGG_13013 and *MoCHS7*, MGG_06064), one β-1,3-glucan synthase gene (*MoFKS1*, MGG_00865) and one α-1,3-glucan synthase gene (*MoAGS2*, MGG_09639), their expression levels were monitored, respectively, by primers (CHS1-QF/CHS1-QR, CHS2-QF/CHS2-QR, CHS3-QF/CHS3-QR, CHS4-QF/CHS4-QR, CHS5-QF/CHS5-QR, CHS6-QF/CHS6-QR and CHS7-QF/CHS7-QR) (Supplementary Table S1). The internal reference gene *MoActin* (MGG_03982) was amplified using the primer pair (Actin-QF/Actin-QR) (Supplementary Table S1) and used to normalize mRNA levels. Relative mRNA expression levels were calculated using the 2^-ΔCt^ method based on the comparative Ct value (Ct = (ΔCT_target_−CT_*Mo*Actin_)). All experiments were conducted in independent biological triplicates with three technical replicates.

## Results

### Sequence and structure analysis of a novel β-1,6-glucanase *Mo*Glu16 from *M. oryzae*

The *MoGLU16* gene contains 1,467 bp and encodes a putative mature protein containing 488 amino acids with pI value of 5.95 and calculated molecular weight of 52 kDa. Signal peptide analysis indicated that *Mo*Glu16 had the N-terminal signal peptide at residues 1–22 (MLKNSILFCLWQAANFYYCVDA), suggesting that it was an extracellular enzyme.

Based on the amino acid sequence alignment of *Mo*Glu16 with other reported endo-1,6-glucanases in the GH5 and GH30 families, a phylogenetic tree of *Mo*Glu16 was created. The results indicated that *Mo*Glu16 was grouped together with the reported β-1,6-glucanases located in the GH30 family, indicating that it was a novel member of the GH30 family. Moreover, *Mo*Glu16 was located in the same branch and shared approximately 50% homology with the β-1,6-glucanases from *A. fumigatus* (EAL85472.1, 50%) and *N. crassa* (BAB_91213.1, 52%), while had about 20% homology with those from *Chitinophaga pinensis* (ACU61803.1, 25%), *S. degradans* (ABD82251.1, 21%), *C. cinerea* (QLF98392.1, 20%) and *L. edodes* (BAK52530.1, 22%).

Multiple alignment of amino acid sequences revealed that *Mo*Glu16 possessed two important amino acid residues, one of which was the acid catalyst residue Glu236 that located on the catalytic box [ITIQNEPL], and the other was the nucleophilic residue Glu332, which were conserved across the reported β-1,6-glucanases from the GH30 family. Moreover, the three-dimensional structure showed *Mo*Glu16 is composed of ten alpha helices and eighteen beta strands; and its overall structure is divided into two domains, the one is the catalytic domain composed of 345 amino acids (76–420), containing the (β/α) barrel fold that made up of five β-strands in its centre and 10 parallel beta-helices around it, as well as the two-stranded mixed β-sheet locates on the C-terminal side of the barrel; the other domain is a β-sandwich domain composed of 143 amino acids (1–75 and 421–488), containing an antiparallel two-strand β-sheets and a mixed eight-stranded β-sheets, both of which were twisted. The substrate-binding sites of *Mo*Glu16 are positioned on top of the β-barrel, which is constituted by 12 amino acid residues (Asp128, Leu129, Trp181, Asn235, Glu236, Asn239, Met244, His305, Tyr307, Glu332, Trp363 and Ala382), among them, most of residues were conserved in other β-1,6-glucanases from GH30. Furthermore, the catalytic residues form a deep U-shaped cleft on the surface of *Mo*Glu16, which may contribute to substrate specificity. In addition, *Mo*Glu16 has some unique amino acids residues near the substrate-binding sites, including Ser180, Ala182, Gln188, Val226, Asp227, Asn241, Gly310, Glu311, Phe329, Gln330, Ser360, Met361 and Phe388.

### Enzymological characteristics of purified recombinant *Mo*Glu16

The recombinant *Mo*Glu16 protein with a 6 × His tag was successfully expressed in *P. pastoris* GS115 and purified using single-step Ni-affinity chromatography. On SDS-PAGE, purified *Mo*Glu16 migrated as a single band with an apparent molecular weight of approximately 52 kDa, which was basically consistent with the predicted molecular weight (52 kDa).

Hydrolase activity of *Mo*Glu16 was measured using various polysaccharides with different glycosidic bonds. *Mo*Glu16 was most active on pustulan at pH 5.0 and 50°C, with a specific activity of 219.0 U/mg (Table 1). Furthermore, it had low activity toward laminarin from *Laminaria digitata*, yeast glucan, and *M. oryzae* cell wall (Table 1). However, *Mo*Glu16 exhibited no hydrolytic activity with pachymaran and xylan from beech wood or corncobs at pH 5.0 and 50°C (Table 1). These results indicated that *Mo*Glu16 was specific for the polysaccharides with continuous β-1,6-glycosidic bonds.

**Table 1:** Substrate specificity of the *Mo*Glu16.

Substrate	Glycoside linkage(s)	Specific activity (U/mg)
Pustulan	β-1,6 (Glc)	219.0^a^ ± 9.42
Laminarin (*Laminaria digitata*)	β-1,3: β-1,6(Glc)	0.308^b^ ± 0.012
Yeast glucan	β-1,3: β-1,6(Glc)	0.285^b^ ± 0.010
*M. oryzae* cell walls	β-Glucan: chitin	0.0023^c^ ± 0.0001
Pachyman	β-1,3 (Glc)	ND
Xylan (beech wood)	β-1,4 (Xyl)	ND
Xylan (corncob)	β-1,4 (Xyl)	ND

ND, not detected.

Different letters are significantly different at *p* ≤ 0.05 according to Duncan’s test.

**Figure 1:**
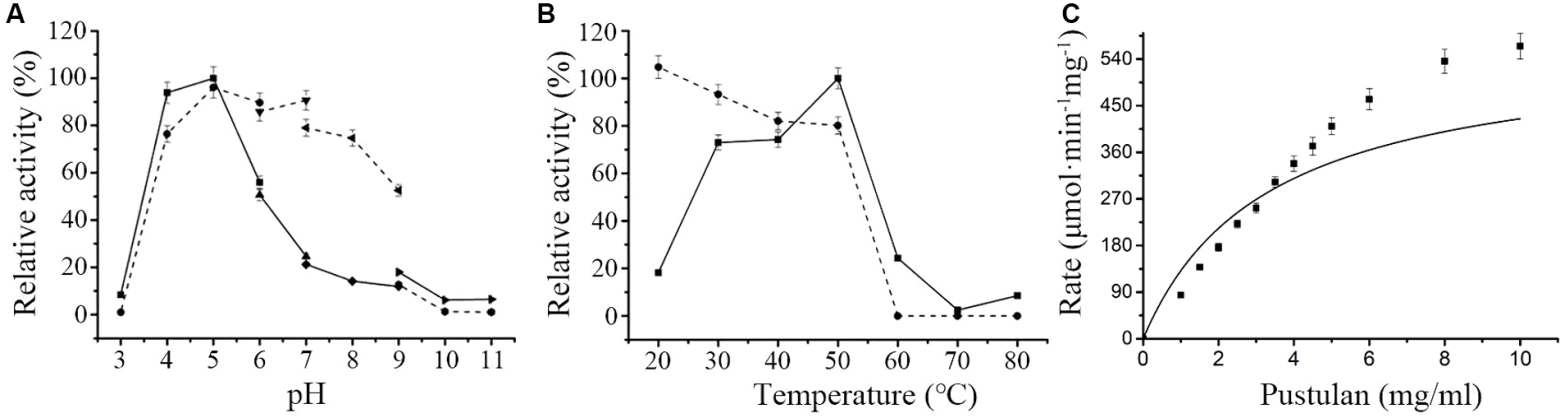
Enzymatic characteristics of *Mo*Glu16. **(A)** Influence of pH on the activity of *Mo*Glu16 with pustulan as the substrate at 50°C. The solid line represented the pH profile and the dotted line represented the pH stability of *Mo*Glu16. **(B)** Impact of temperature on the activity of *Mo*Glu16 with pustulan as the substrate at pH 5.0. The solid line represented the temperature profile and the dotted line represented the thermostability of *Mo*Glu16. **(C)** Effect of the substrate concentration of pustulan on the hydrolytic activity of *Mo*Glu16. Bars show the standard deviations of the averages from three replicates.

Maximal hydrolytic activity of *Mo*Glu16 toward pustulan was obtained in citrate buffer at pH 5.0 (Figure 1A). Moreover, *Mo*Glu16 retained over 65% of the maximal activity after incubation at 4°C in the pH range of 4.0–8.0 for 12 h, indicating it had good pH stability (Figure 1A). Furthermore, at pH 5.0, the maximal activity of *Mo*Glu16 was attained at 50°C, while after incubation for 1 h at 20–50°C, *Mo*Glu16 retained over 75% of the highest hydrolytic activity (Figure 1B). In addition, the effect of pustulan with different concentrations on the hydrolytic capacity of *Mo*Glu16 was also investigated. As the concentration was less than 4 mg/mL, the hydrolytic activity of *Mo*Glu16 enhanced with increasing substrate concentration. The K_m_ was calculated to be 3.30 μM and V_max_ was 564.79 mΜ·min^−1^·mg protein^−1^ (Figure 1C).

In addition, we also investigated the effects of metal ions and EDTA on the hydrolytic activity of *Mo*Glu16. The addition of Fe^2+^, Na^+^, Cu^2+^ and Mg^2+^ enhanced the activity of *Mo*Glu16 by 15.7, 36.2, 36.2, and 49.5%, respectively (Table 2). By contrast, Zn^2+^ strongly inhibited the activity of *Mo*Glu16, by up to 86.4%. In addition, the presence of 1 mM EDTA inhibited approximately 47.3% the enzyme activity, while 10 mM EDTA completely inhibited the activity of *Mo*Glu16 (Table 2). These results indicated that *Mo*Glu16 is a metalloenzyme.

**Table 2:** Effects of metal ions and the chelator EDTA on the hydrolytic activity of *Mo*Glu16 against pustulan.

Reagent	Concentration (mM)	Relative activity (%)
None	1	100^b^ ± 4.13
K^+^ (KCl)	1	63.93^e^ ± 2.56
Na^+^ (NaCl)	1	136.17^a^ ± 5.45
Ba^2+^ (BaCl_2_)	1	77.90^c^ ± 3.12
Co^2+^ (CoCl_2_)	1	72.23^d^ ± 2.89
Cu^2+^ (CuCl_2_)	1	136.16^a^ ± 5.45
Fe^2+^ (FeCl_2_)	1	115.72^b^ ± 4.63
Mg^2+^ (MgCl_2_)	1	149.52^a^ ± 5.98
Mn^2+^ (MnCl_2_)	1	66.81^e^ ± 2.67
Ni^2+^ (NiCl_2_)	1	71.97^d^ ± 2.88
Zn^2+^ (ZnCl_2_)	1	13.62^g^ ± 0.54
Cr^3+^ (CrCl_3_)	1	89.17^c^ ± 3.57
EDTA	1	52.66^f^ ± 2.11
EDTA	10	0^h^

Different letters are significantly different at *p* ≤ 0.05 according to Duncan’s test.

### *Mo*Glu16 hydrolyzed pustulan into oligosaccharides with degrees of polymerization of 1–6

**Figure 2:**
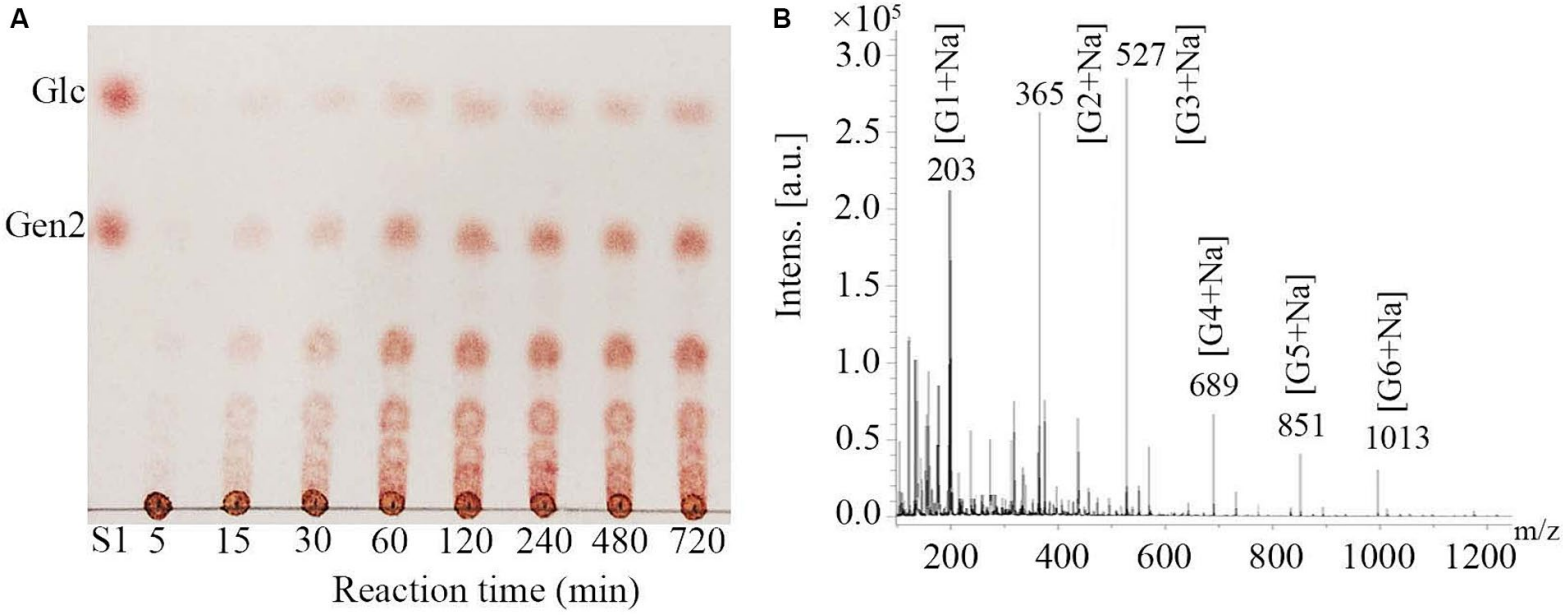
Analysis of the hydrolysates after the action of *Mo*Glu16 to pustulan. **(A)** TLC analysis of the hydrolysates produced at different incubation time (5 min, 15 min, 30 min, 1 h, 2 h, 4 h, 8 h and 12 h). **(B)** MALDI-TOF MS spectra of the reaction mixture of pustulan and *Mo*Glu16 at 12 h.

Hydrolysates of pustulan produced by *Mo*Glu16 were analyzed using thin layer chromatography (TLC). As illustrated in Figure 2A, *Mo*Glu16 hydrolyzed pustulan into a series of oligosaccharides and the products accumulated gradually with the prolongation of reaction time. In addition, the degrees of polymerization of the products generated by hydrolyzing pustulan with *Mo*Glu16 for 12 h were assessed. MALDI-TOF MS analysis of the hydrolysates showed that six fragment ions appeared at m/z 203, m/z 365, m/z 527, m/z 689, m/z 851 and m/z 1,013, respectively (Figure 2B), corresponding to the molecular weights of [G1 + Na] (G1, glucose), [G2 + Na] (G2, disaccharide), [G3 + Na] (G3, trisaccharide), [G4 + Na] (G4, tetrasaccharide), [G5 + Na] (G5, pentasaccharide) and [G6 + Na] (G6, hexasaccharide), respectively. In addition, *Mo*Glu16 did not act on gentiobiose (data not shown). These results revealed that *Mo*Glu16 cleaved pustulan into oligosaccharides possessing degrees of polymerization from 1 to 6 via an endo-type mechanism.

### Distribution of β-1,6-glucan in mycelia, conidia and bud tubes of *Magnaporthe oryzae*

**Figure 3:**
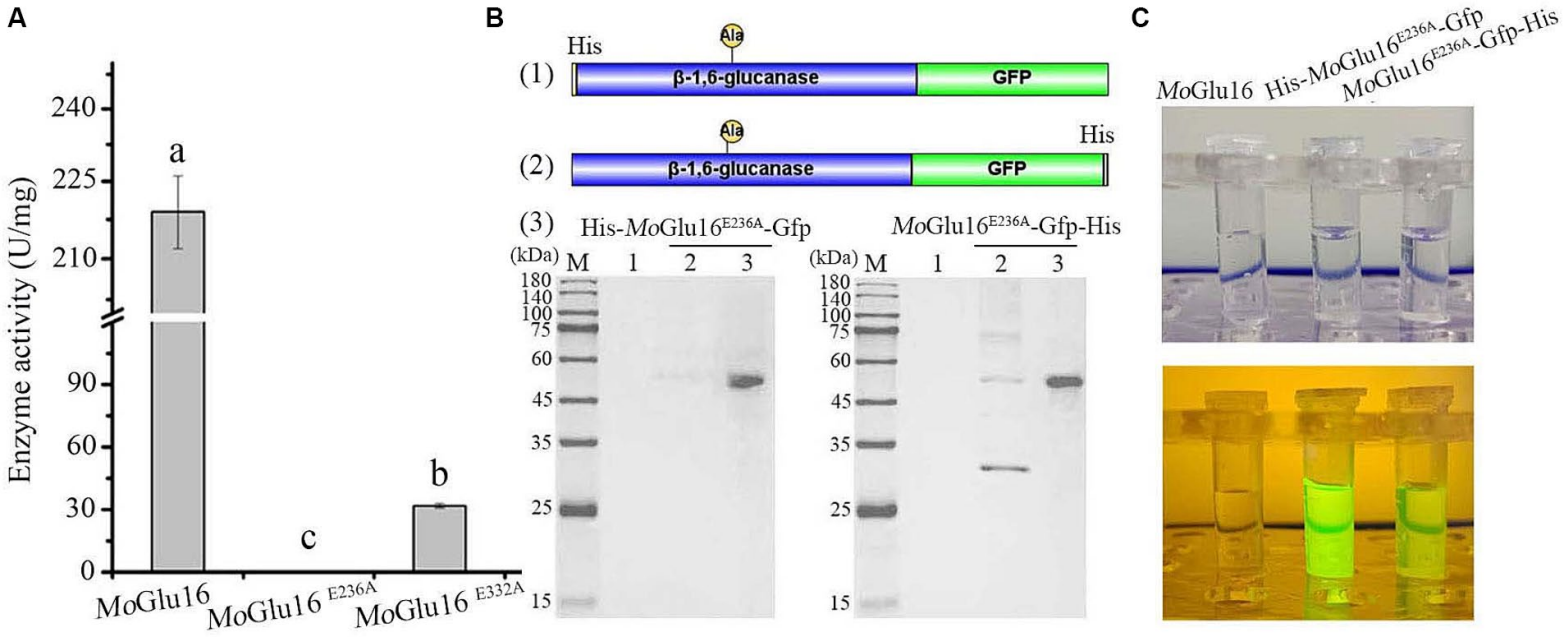
Construction of the fluorescent derivatives derived from *Mo*Glu16 without hydrolytic activity. **(A)** Hydrolytic activity analysis of *Mo*Glu16 and its derivatives (*Mo*Glu16^E236A^ and *Mo*Glu16^E332A^) to pustulan. Bars indicate the standard deviations of the averages from three replicates. Columns with different letters are significantly different at *p* ≤ 0.05 according to Duncan’s test. **(B)** Construction and expression of the different fluorescent derivatives. (1) and (2), the construction of the derived proteins His-*Mo*Glu16^E236A^-Gfp and *Mo*Glu16^E236A^-Gfp-His, respectively; (3), the expression of the derivatives by SDS-PAGE detection. **(C)** Fluorescence detection of *Mo*Glu16 and the derivatives (His-*Mo*Glu16^E236A^-Gfp and *Mo*Glu16^E236A^-Gfp-His) under blue light.

Given the specific binding of *Mo*Glu16 to the substrate, β-1,6-glucan in the cell wall was specifically detected using an inactivated mutant of *Mo*Glu16. Mutation of the amino acids residue Glu236 resulted in the loss of the hydrolytic activity of *Mo*Glu16 toward pustulan (Figure 3A). To visualize the binding of *Mo*Glu16^E236A^ to the substrate, we constructed the GFP-tagged variants His-*Mo*Glu16^E236A^-Gfp and *Mo*Glu16^E236A^-Gfp-His, respectively (Figure 3B). Compared with *Mo*Glu16^E236A^-Gfp-His, the same amount of His-*Mo*Glu16^E236A^-Gfp showed stronger fluorescence when the protein was exposed to blue light (Figure 3C).

**Figure 4:**
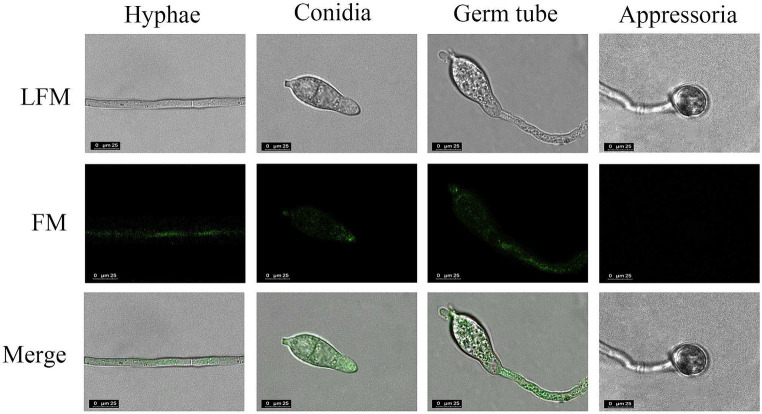
Localization of β-1,6-glucan in different development stages of *M. oryzae*. The cells of *M. oryzae* (hyphae, conidia, germ tube and appressoria) were incubated, respectively, with His-*Mo*Glu16^E236A^-Gfp for 1 h at 28°C, and then washed with 20 mM Tris–HCl buffer. Finally, the cells were observed under fluorescence microscope. LFM, light-field microscope; FM, fluorescence microscope. All images were observed at a magnification of 63 under confocal laser scanning microscopy (CLSM) (CLSM, Leisa, TCSSP2). The results represent one of three replicates with similar results.

Next, the specificity of His-*Mo*Glu16^E236A^-Gfp for the insoluble polysaccharides with different glycosidic bonds (pustulan, β-1,6-glucan; yeast glucan, β-1,3;1,6-glucan; pachyman, β-1,3-glucan) was determined. After incubation of His-*Mo*Glu16 ^E236A^-Gfp with the above three polysaccharides, green fluorescence was observed with pustulan and yeast glucan, whereby the former gave a strong fluorescence signal; while no fluorescence was observed with pachyman. However, no fluorescence present as the incubation of the GFP protein with the above insoluble polysaccharides. These results illustrated binding specificity of His-*Mo*Glu16^E236A^-Gfp for polysaccharides containing continuous β-1,6-glucoside bonds. Based on this, β-1,6-glucan in the cell wall of *M. oryzae* cell wall was specifically visualized using His-*Mo*Glu16 ^E236A^-Gfp. Green fluorescence was observed on mycelia, conidial tips and bud tubes, but not on appressoria (Figure 4), revealing that β-1,6-glucan was distributed in the vegetative hyphae, conidia and bud tubes of *M. oryzae*.

### High doses of *Mo*Glu16 inhibited conidia germination and appressorium formation of *Magnaporthe oryzae*

**Figure 5:**
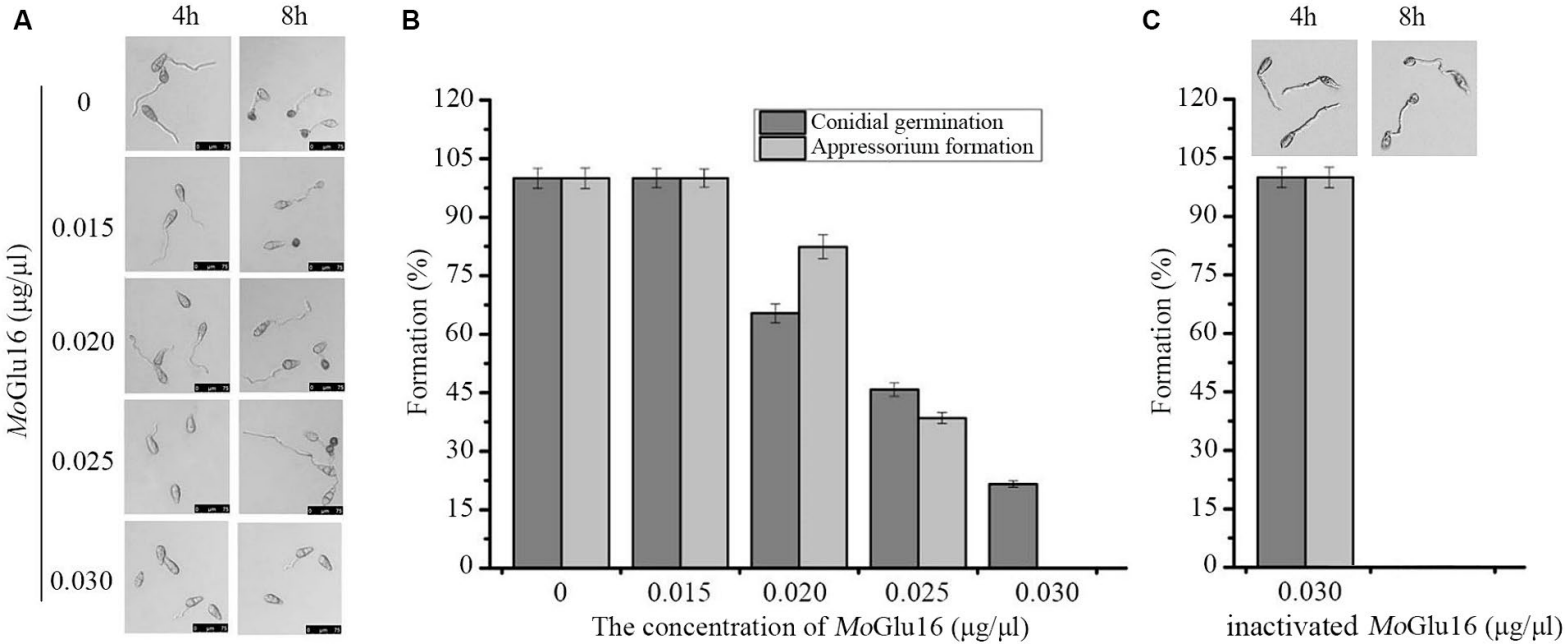
Effect of *Mo*Glu16 on conidial development. **(A)** Assay of conidial germination treated by various concentrations of *Mo*Glu16. Conidial germination (4 h) and appressorium formation (8 h) were observed by differential interference contrast (DIC) microscope. **(B)** Quantitative analysis of the conidial germination and the appressorium formation. **(C)** Assay of conidial germination treated by 0.03 μg/μl inactivated *Mo*Glu16. Bars indicate the standard deviations of the averages from three replicates.

Conidial germination and appressorium formation are vital processes during the infection of rice by *M. oryzae*, during which the structure of the cell wall is remolded. Beta-1,6-glucan is crucial in maintaining the structural integrity of the cell wall, so we decided to study the effect of *Mo*Glu16 on conidial development of *M. oryzae*. As shown in Figure 5, *Mo*Glu16 prominently inhibited germ tube germination and appressorium formation in a dose-dependent manner. Furthermore, the lowest concentration of *Mo*Glu16 inhibiting conidial germination was 0.02 μg/μl. When the concentration was increased to 0.03 μg/μl, appressorium formation was completely inhibited (Figures 5A,B). By contrast, it had no effect on conidial germination and appressorium formation after treated by inactivated *Mo*Glu16 (0.03 μg/μl) (Figure 5C), suggesting that *Mo*Glu16 participated in the remodeling of cell walls during conidial development of *M. oryzae*.

### Treatment with *Mo*Glu16 led to the accumulation of ROS in the mycelium of *Magnaporthe oryzae*

**Figure 6:**
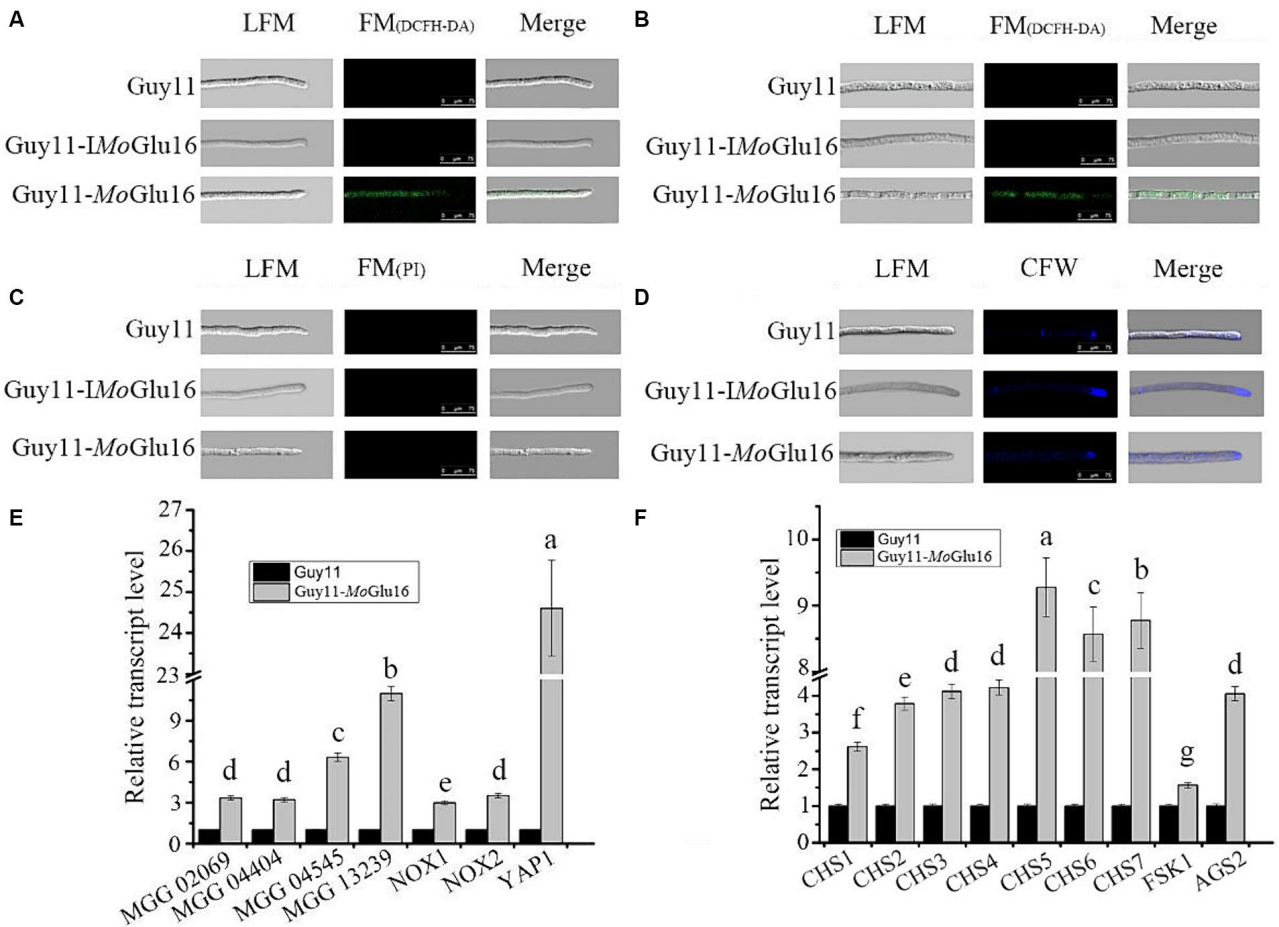
Effects of *Mo*Glu16 on mycelial growth of *M. oryzae*. **(A,B)** Detection of ROS accumulation in mycelial apical **(A)** and median **(B)** based on H_2_DCFDA staining after treatment with *Mo*Glu16 for 12 h. LFM, light-field microscope; FM, fluorescence microscope; Guy11, untreated *M. oryzae*; Guy11-I*Mo*Glu16, *M. oryzae* treated by 0.30 μg/mL inactivatd *Mo*Glu16; Guy11-*Mo*Glu16, *M. oryzae* treated by 0.30 μg/mL *Mo*Glu16. **(C)** Detection of cell membrane integrity of mycelium via PI staining after treatment with *Mo*Glu16 for 12 h. **(D)** Detection of chitin in the cell wall of mycelium treated by *Mo*Glu16 for 12 h. **(E)** Expression levels of genes related to reactive oxygen species production. These genes contained glyoxalase gene (MGG_02069), peroxidase genes (MGG_04404, MGG_04545 and MGG_13239), superoxide-generating NADPH-oxidase genes (*NOX1*, MGG_00750; and *NOX2*, MGG_06599) and oxidative stress regulator gene (*YAP1*, MGG_12814). **(F)** Expression levels of genes correlated with cell wall polysaccharide synthases. These genes included chitin synthase genes (*CHS1*, MGG_01802; *CHS2*, MGG_04245; *CHS3*, MGG_09551; *CHS4*, MGG_09962; *CHS5*, MGG_13014; *CHS6*, MGG_13013; and *CHS7*, MGG_06064), β-1,3-glucan synthase gene *FKS1* (MGG_00865) and α-1,3-glucan synthase gene *AGS2* (MGG_09639). The values represent the means of the three replicates with standard deviation (error bars). Columns with different letters are significantly different at *p* ≤ 0.05 according to Duncan’s test.

The effect of *Mo*Glu16 on vegetative mycelia of *M. oryzae* was assessed by detecting the generation of ROS and the distribution of chitin. ROS can activate the cell wall integrity pathway (CWI) and further induce cell wall remolding. In the presence of ROS, H_2_DCFDA diffuses into the cell and is oxidized into the green fluorescent chromophore DCF (2′,7′-dichlorofluorescein) (Munkres, 1990). Green fluorescence was observed in the apical (Figure 6A) and median sections (Figure 6B) of the hyphae treated with *Mo*Glu16 for 12 h. Since a previous study reported that excessive ROS accumulation in mycelia leads to cell death (Petrov et al., 2015), the condition of mycelia was further examined via PI staining. No red fluorescence was found in the mycelia incubated with or without *Mo*Glu16 (Figure 6C). In addition, the colonies with the same size were found when the mycelia treated by *Mo*Glu16 or inactivated *Mo*Glu16 were cultured on CM solid medium for 5 days at 28°C, indicating that the treatment with *Mo*Glu16 did not trigger cell death. To further investigate the underlying mechanism, the distribution of chitin in the treated mycelia was detected. Blue fluorescence was observed in the apices and septa of the mycelia with or without *Mo*Glu16 (Figure 6D), suggesting that treatment with *Mo*Glu16 had no effect on the distribution of chitin.

In addition to the phenotypic experiments, we explored the expression of genes involved in ROS generation and cell wall polysaccharide synthesis in the *Mo*Glu16-treated mycelia. As shown in Figure 6E, treatment with *Mo*Glu16 resulted in the up-regulation in expression level of genes related to ROS synthesis. Among them, the expression level of oxidative stress regulator gene (*YAP1*, MGG_12814) was significantly enhanced, with a 24.6-fold increase in *Mo*Glu16-treated mycelia (Figure 6E). In addition, the expression levels of genes involved in the synthesis of cell wall polysaccharides were also assessed. The expression levels of seven chitin synthase genes (*MoCHS1-MoCHS7*) were increased in the *Mo*Glu16-treated mycelia. Among them, *MoCHS5*, *MoCHS6* and *MoCHS7* were expressed at higher levels, with 9.3-, 8.6- and 8.8-fold increases, respectively (Figure 6F). Similarly, the transcript level of the α-1,3-glucan synthase gene *MoAGS2* (MGG_09639) was increased approximately 3.8-fold (Figure 6F); while the transcript level of the β-1,3-glucan synthase gene *MoFKS1* (MGG_00865) was only slightly elevated (Figure 6F). These results indicated that the external addition of *Mo*Glu16 disturbed the balance between synthesis and hydrolysis during mycelia cell wall remodeling, causing intracellular ROS accumulation and further activating the cell wall integrity pathway, resulting in the up-regulation in expression level of genes involved in the synthesis of cell wall polysaccharide.

## Discussion

An endo-β-1,6-glucanase *Mo*Glu16 was cloned from *M. oryzae* and found to be a novel member of glycoside hydrolase family 30 (GH30). *Mo*Glu16 not only had the typical characteristic of β-1,6-glucanases from GH30, such as the conserved catalytic motif, invariant substrate-binding sites and two-domains protein structure, but also possessed the unique features, such as the distinctive amino acids near the substrate-binding sites, including Gln188, Glu311, Gln330 and Phe388, which may be contributed to the difference in enzymatic characteristics between *Mo*Glu16 and other β-1,6-glucanases from GH30. In this work, *Mo*Glu16 cleaved β-1,6-glycosidic bonds of pustulan and produced a series of oligomers with degrees of polymerization (DP) of 1–6, exhibiting strict substrate specificity. At present, the reported β-1,6-glucanases had molecular weights between 40 and 60 kDa, optimal temperatures from 40 to 50°C, and an optimal pH between 5.0 and 7.0; furthermore, all of them exhibited high hydrolytic activity toward pustulan, as shown in Table 3. Although having the same optimal substrate, they showed diverse hydrolytic efficiency. In comparison with the reported β-1,6-glucanases, the activity of *Mo*Glu16 toward pustulan was 219 U/mg, second only to that of β-1,6-glucanases from *N. crassa* (803.5 U/mg) (Hiura et al., 1987) and *C. cinerea* (776.5 U/mg) (Liu et al., 2020). Compared to the oligomers (DP1-6) produced by *Mo*Glu16, other β-1,6-glucanases generated oligomers with DP of less than 6, such as those from *S. degradans* (Gly30B, DP 1–2) (Wang et al., 2017) and *T. harzianum* (BGN16.3, DP 1–5; BGN16.1, DP 1–4; and BGN16.2, DP 2–5) (de la Cruz and Llobell, 1999; Montero et al., 2005), which may be arise from the differences of substrate binding grooves between *Mo*Glu16 and other β-1,6-glucanases.

**Table 3:** Comparison of properties of the *Mo*Glu16 and other reported β-1,6-glucanases.

Microorganism	β-1,6-glucanases	Molecular mass (kDa)	Optimal temperature (°C)	Optimal pH	Specific activity (U/mg)	Optimal subtrate	End products	Reference or source
*Magnaporthe oryzae*	*Mo*Glu16	54	50	5	219	Pustulan	DP1-6	This paper
*Neurospora crassa*	β-1,6-glucanase	47	50	5.5	803.5	Pustulan	oligomer	Hiura et al. (1987)
*Coprinopsis cinerea*	GH30A	56	60	6	776.5	Pustulan	oligomer	Liu et al. (2020)
*Trichoderma harzianum*	BGN16.3	46	50	5	188	Pustulan	DP1-5	Montero et al. (2005)
*Saccharophagus degradans*	Gly30B	53	40	7	153.8	Pustulan	DP1-2	Wang et al. (2017)
*Trichoderma harzianum*	BGN16.1	51	50	5.5	150	Pustulan	DP1-4	de la Cruz and Llobell (1999)
*Trichoderma harzianum*	BGN16.2	43	50	5.5	86	Pustulan	DP2-5	de la Cruz et al. (1995)
*Lentinula edode*	LePus30A	49	50	5	14.8	Pustulan	oligomer	Konno and Sakamoto (2011)
*Neurospora crassa*	Neg1	50	40	5	13.4	Pustulan	ND	Oyama et al. (2002)
*Trichoderma virens*	Tvbgn3	48	40	5	4.2	Pustulan	ND	Djonovic et al. (2006)
*Neotyphodium* sp.	β-1,6-glucanase	47	40	5.5	0.8	Pustulan	ND	Moy et al. (2002)

Based on the specific binding of *Mo*Glu16 to the polysaccharides containing the continuous β-1,6-glucoside bond, a new simple method was developed to detect the distribution of β-1,6-glucan in the cell wall of *M. oryzae*. Currently, the molecular probes using to detect α-glucan, β-1,3-glucan and chitin of fungal cell wall were commercially available, such as the fluorophore-labeled antibodies monitoring the α-glucan (IgMg MoPC-104E) and β-1,3-glucan (Mouse IgG Kappa Light), and the fluorophore-labeled lectin detecting chitin (WGA-Alexa532) (Fujikawa et al., 2012), while commercial antibodies enabling to test β-1,6-glucan were not available. The methods using to detect β-1,6-glucan have only been described in a few papers. Oliveira-Garcia and Deising (2016) reported a chimeric protein β-1,6-GBD: YFP, consisting of the β-1,6-glucan-binging domain of endo-β-1,6-glucanase of *C. graminicola* and the YFP of *Aequorea Victoria*, and applied it to detect β-1,6-glucan in the infection structure of *C. graminicola*, but further investigation will be solved about whether the protein β-1,6-GBD: YFP is species-specific and whether it is applicable to other filamentous ascomycetes. In addition, Yamanaka et al. (2020) reported the system to detect β-1,6-glucan by the *Neurspora-*derived endo-β-1,6-glucanase without hydrolase activity, in which extracellular β-1,6-glucan released by *Candida* species and β-1,6-glucan exposed on the cell wall in yeast forms of *Candida* were assayed by flow cytometer. The single cell was quickly analyzed by flow cytometer, however, it wasn’t suitable for the ascomycetes in filamentous form. In our work, distribution of β-1,6-glucan on cell wall in rice blast was detected by the *M. oryzae*-derived recombinant β-1,6-glucanase *Mo*Glu16 without hydrolytic activity. Evidence for the presence of β-1,6-glucan in the cell wall of blast rice has previously been reported in other studies, for example, Li et al. (2019) found the cell wall of rice blast changed from the tight arrangement to the loose saccule after treatment with the β-1,6-glucanase GluM from *Corallococcus* sp. strain EGB, moreover, the hyphae were transformed into a grid-like pattern and conidia were in a shrunken state after GluM hydrolysis, while no obvious phenomenon occurred in the fungi without β-1,6-glucans in their cell wall, suggesting β-1,6-glucan was the essential component in cell wall of *M. oryzae*. In addition, our study on detection for β-1,6-glucan by the inactivated GFP-tagged protein (His-*Mo*Glu16^E236A^-Gfp) provided more direct evidence for the presence of β-1,6-glucan in cell wall of blast fungus, however, the further investigation on the content of each polysaccharides component and the structure of cell wall will be required in the future.

β-1,6-glucan may serve as an excellent antifungal target in the biological control of rice blasts. As a flexible glue in the formation of covalent cross-links, β-1,6-glucan enables to interconnect other cell wall components, which is crucial for the growth of fungal cells. The deletion of genes involved in β-1,6-glucan synthesis or the inhibition of corresponding biosynthetic enzyme activity leads to the severe defects of cell wall and the decrease or loss of infection ability in fungal pathogens (Oliveira-Garcia and Deising, 2016; Han et al., 2019). Kuroyanagi et al. (2010) reported that triazolopyridines, as an inhibitor of β-1,6-glucan synthesis, exhibited potent antifungal activity, suggesting proteins involved in the synthesis of β-1,6-glucan were the worthy antifungal target in plant disease control. Besides that, as the glucoside hydrolases specifically cleaving β-1,6-glycosidic bonds, β-1,6-glucanases may be potential application in plant fungal disease protection. In this study, β-1,6-glucan in the hyphae, conidia, germ tubes and appressoria of *M. oryzae* was detected by utilizing specificity of the β-1,6-glucanase *Mo*Glu16 binding to substrate, suggesting it may be as a potential candidate for antifungal targets. Moreover, excess *Mo*Glu16 triggered the generation of reactive oxygen species (ROS) in the mycelia, which may activate the of cell wall integrity pathway and further cause expression of genes involved in cell wall polysaccharides synthesis. Furthermore, excess *Mo*Glu16 prominently inhibited the conidial development of *M. oryzae* and affected the appressorial formation, indicating β-1,6-glucanase participated in fungal cell wall reconstruction, providing the important foundation for the creating of the disease-resistant transgenic plants. In addition, the only presence of β-1,6-glucan is in fungal cell wall and some members of oomycetes, while it is absent in plant cell wall and bacteria (Fesel and Zuccaro, 2016), suggesting the transgenic plants harboring genes encoding β-1,6-glucanase may show highly selective attack against fungi. In conclusion, this study not only provides the novel antifungal target for the development of the green and reliable biocontrol agents, but also offers the valuable genetic resources for the breeding of resistant varieties.

## Data availability statement

The original contributions presented in the study are included in the article/Supplementary material, further inquiries can be directed to the corresponding authors.

## Author contributions

YW: Conceptualization, Funding acquisition, Writing – original draft, Writing – review & editing. DL: Funding acquisition, Methodology, Software, Writing – original draft. ZL: Funding acquisition, Methodology, Project administration, Writing – review & editing. ZC: Funding acquisition, Project administration, Writing – review & editing. XY: Conceptualization, Funding acquisition, Writing – review & editing.

## Footnotes

^1^ http://zhanglab.ccmb.med.umich.edu/I-TASSER
