# Supplementary material for: Functional analysis of a novel endo-β-1,6-glucanase MoGlu16 and its application in detecting cell wall β-1,6-glucan of Magnaporthe oryzae
Source: Front Microbiol. 2024 Jul 4;15:1429065. doi: 10.3389/fmicb.2024.1429065 (PMC11254853; doi:10.3389/fmicb.2024.1429065)
Supplement: Supplementary file 1 [file Data_Sheet_1.docx]

**Supplementary Material**

**Functional analysis of a novel endo-β-1,6-glucanase *Mo*Glu16 and its application in detecting cell wall β-1,6-glucan of *Magnaporthe oryzae***

Yanxin Wang ^1, 2^, Ding Li ^3^, Zhoukun Li ^2^, Zhongli Cui ^2^, Xianfeng Ye ^2*^

1 College of Life Sciences of Liaocheng University, 252000 Liaocheng, P. R. China

2 Key Laboratory of Agricultural Environmental Microbiology, Ministry of Agriculture and Rural Affairs, College of Life Sciences of Nanjing Agricultural University, 210095 Nanjing, P. R. China

3 Institute of Veterinary Immunology & Engineering, Jiangsu Academy of Agricultural Sciences, 210014 Nanjing, P. R. China

§ The corresponding author

To whom correspondence should be addressed: Dr. Xianfeng Ye

*E-mail: [yxf@njau.edu.cn](mailto:yxf@njau.edu.cn) or 17805003810@163.com

**Table S1** Primer sets used in this study

| Name | Primer | Sequences (5'-3') of primers used | Remark |
| --- | --- | --- | --- |
| *MoGLU16* | *Mo*Glu16-F | AGAAGAAGGGGTATCTCTCGAGATGGTCAACGTGCCTCGAAAAGAAG | Expression of the protein *Mo*Glu16 |
|  | *Mo*Glu16-R | GAGATGAGTTTTTGTTCTAGATTAGTGGTGGTGGTGGTGGTGGGCCGGGGGCAGGAGCCAGGTA |  |
| E236A | E236A-F | AATCCAAAACGCACCCCTCAACAGCAATGCCGG | Expression of *Mo*Glu16 ^E236A^ |
|  | E236A-R | AGGGGTGCGTTTTGGATTGTAATGGCATCGACA |  |
| E332A | E332A-F | ATTCCAGACCGCGTGTTGGACGTCTGCAAAGCA | Expression of *Mo*Glu16 ^E332A^ |
|  | E332A-R | ATTCCAGACCGCGTGTTGGACGTCTGCAAAGCA |  |
| His-N-GFP | N-His-F1 | GAAGGGGTATCTCTCGAGATGCATCATCATCATCATCATTTGAAAAATTCGATTCTGTT | Expression of His-MoGlu16 ^E236A^-Gfp |
|  | NC-His-R1 | AGCTCCTCGCCCTTGCTCACGGCCGGGGGCAGGAGCCA |  |
|  | NC-His-F2 | GTGAGCAAGGGCGAGGAGC |  |
|  | N-His-R2 | GAGATGAGTTTTTGTTCTAGATTACTTGTACAGCTCGTCCATGCC |  |
| C-His-GFP | C-His-F1 | GAAGAAGGGGTATCTCTCGAGATGTTGAAAAATTCGATTCTGTTCTG | Expression of MoGlu16 ^E236A^-Gfp-His |
|  | NC-His-R1 | AGCTCCTCGCCCTTGCTCACGGCCGGGGGCAGGAGCCA |  |
|  | NC-His-F2 | GTGAGCAAGGGCGAGGAGC |  |
|  | C-His-R2 | ATGAGTTTTTGTTCTAGATTAGTGGTGGTGGTGGTGGTGCTTGTACAGCTCGTCCATGC |  |
| Factor | 5-α-factor | ATGAGATTTCCTTCAATTTTTACTGC | Verification of yeast transformation strains |
| AOX | 3-AOX | GGCAAATGGCATTCTGACAT |  |
| *MoActin* | Actin-QF | GACCGACTACCTGATGAAGA | Quantiative RT-PCR analysis of *Mo*Actin |
|  | Actin-QR | TGCCGATGGTGATAACCTG |  |
| *Mo*Glu16 | *Mo*Glu16-QF | CCCCTGAATACACCTATGATG | Quantiative RT-PCR analysis of *Mo*Glu16 |
|  | *Mo*Glu16-QR | TCCTCGGGAAAGACATAAAGACTGG |  |
| MGG_02069 | 020-QF | ACAACGGTCGCCTTTTACAC | Quantiative RT-PCR analysis |
|  | 020-QR | GTGTTGGTGTTGGCCTTTTC |  |
| MGG_04404 | 044-QF | CCACGAGCTCAACTTTGGAT |  |
|  | 044-QR | GGACGGTGACAAGCATCTCT |  |
| MGG_04545 | 045-QF | CTGCTGCTCAACGAGAAGTG |  |
|  | 045-QR | ACACCGAGCTCAAACAGCTT |  |
| MGG_13239 | 132-QF | GTCACAGCGTGACGACAAGT |  |
|  | 132-QR | GTCCTCCTGTCGCTTCAGAC |  |
| *NOX1* | NOX1-QF | ACATGGACACCACCCAGAAC |  |
| (MGG_00750) | NOX1-QR | CATGCTTCCCTCGAGACCAC |  |
| *NOX2* | NOX2-QF | ATCAAGGCCGATGATGCTAC |  |
| (MGG_06599) | NOX2-QR | ATGTGCTACCCAGACCCTTG |  |
| *YAP1* | YAP1-QF | AGGTGCAGAACGGCGACTTCGAT |  |
| (MGG_12814) | YAP1-QR | CTAGCTCGACGTCGCCGCACCT |  |
| *MoCHS1* | CHS1-QF | TCAACGACGAGGAGAAGCC | Quantiative RT-PCR analysis of *Mo*CHS1 |
|  | CHS1-QR | GTAATCGCAACAGCCAAGA |  |
| *MoCHS2* | CHS2-QF | TCCACGACCTTTGCCATCA | Quantiative RT-PCR analysis of *Mo*CHS2 |
|  | CHS2-QR | CGCTTTTGCTTCCGCGACT |  |
| *MoCHS3* | CHS3-QF | CGGAAACCAAGGAACAGCG | Quantiative RT-PCR analysis of *Mo*CHS3 |
|  | CHS3-QR | CAGGGAACAACCAAGAACCAC |  |
| *MoCHS4* | CHS4-QF | TCGAGGGAAAATGTAACGG | Quantiative RT-PCR analysis of *Mo*CHS4 |
|  | CHS4-QR | TACTGCTGCTGGTGATGGT |  |
| *MoCHS5* | CHS5-QF | CCGTGTTGATGGAGGTTGA | Quantiative RT-PCR analysis of *Mo*CHS5 |
|  | CHS5-QR | GATCTGGCGGTCGAGGAAT |  |
| *MoCHS6* | CHS6-QF | GAACGGCAGATTTGATGAC | Quantiative RT-PCR analysis of *Mo*CHS6 |
|  | CHS6-QR | ACAAGAGTGCTTCGGTGGC |  |
| *MoCHS7* | CHS7-QF | GACATTGAGCTGGAGATTGG | Quantiative RT-PCR analysis of *Mo*CHS7 |
|  | CHS7-QR | CGCCGCTGTTGCTGTTGTT |  |
| *MoFSK1* | FSK1-QF | TGGCATACAATTTCGCAGCCGG | Quantiative RT-PCR analysis of *Mo*FSK1 |
|  | FSK1-QR | TTGTTATGGCCTTTGGTGG |  |
| *MoAGS2* | AGS2-QF | GACCGACGATGAGTTTCTGC | Quantiative RT-PCR analysis of *Mo*AGS2 |
|  | AGS2-QR | AGAACCAGATGACCGAGGTG |  |
